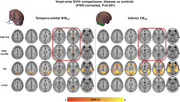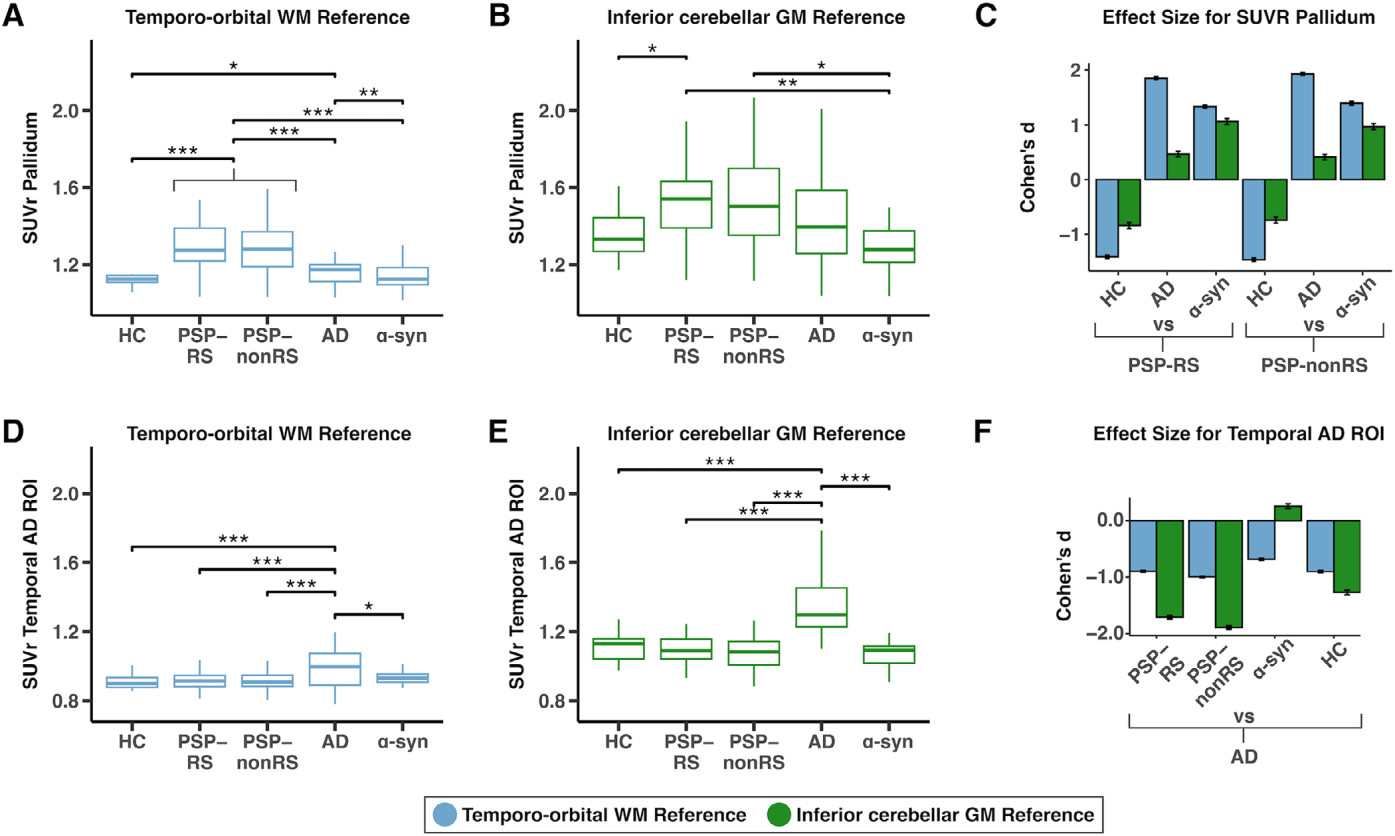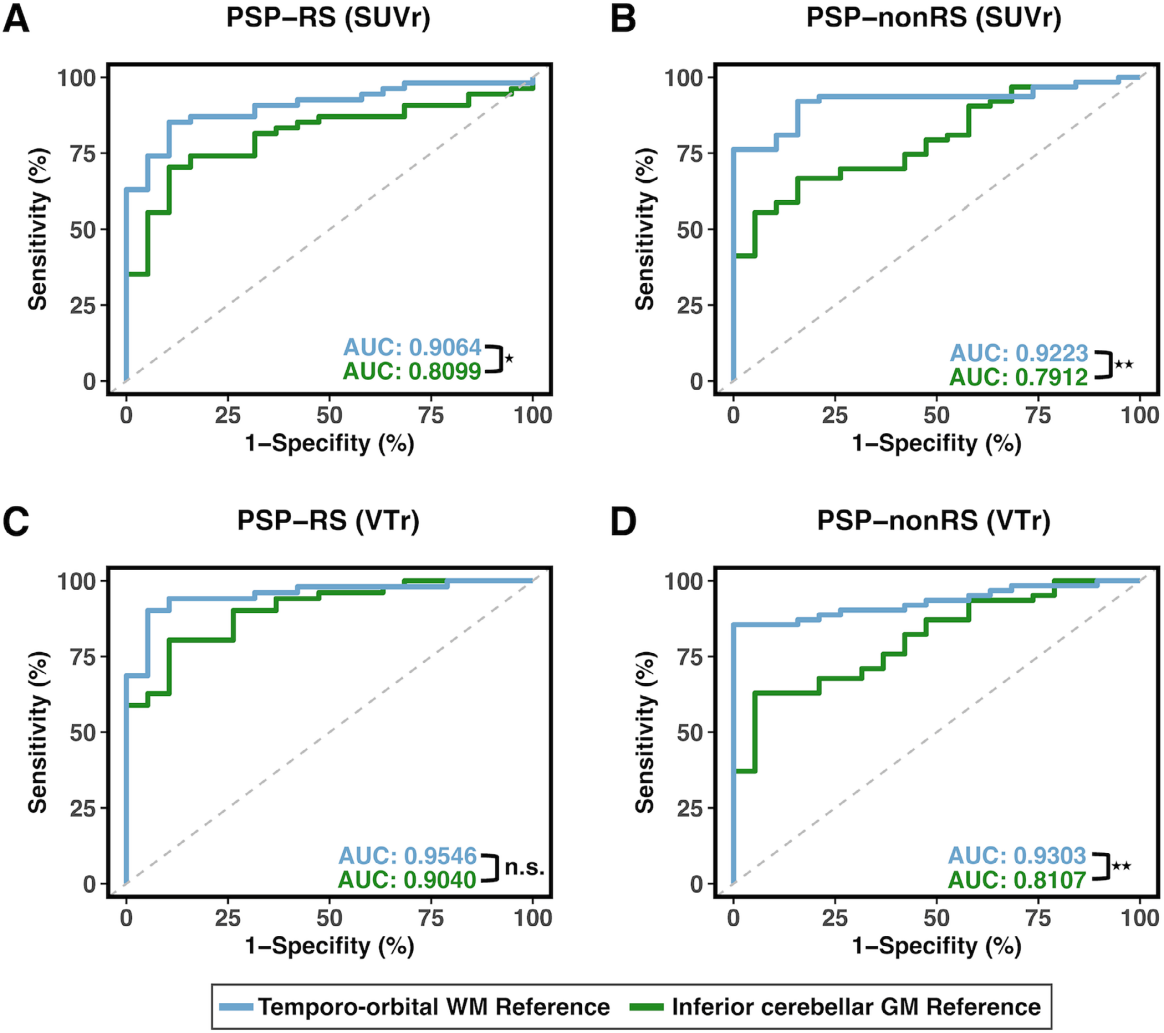# Developing a Novel Reference Region for PI‐2620‐PET Imaging to Facilitate Assessment of 4‐Repeat Tauopathies

**DOI:** 10.1002/alz70856_104777

**Published:** 2026-01-07

**Authors:** Lukas Frontzkowski, Mattes Gross, Sebastian Roemer‐Cassiano, Carla Palleis, Amir Dehsarvi, Sabrina Katzdobler, Anna Dewenter, Anna Steward, Davina Biel, Fabian Hirsch, Johannes Gnörich, Johannes Levin, Andrew W. Stephens, Andre Mueller, Norman Koglin, Gérard N Bischof, Gabor G. Kovacs, Günter U Höglinger, Matthias Brendel, Nicolai Franzmeier

**Affiliations:** ^1^ Institute for Stroke and Dementia Research (ISD), University Hospital, LMU Munich, Munich, Bavaria, Germany; ^2^ Department of Nuclear Medicine, University Hospital, LMU Munich, Munich, Bavaria, Germany; ^3^ Institute for Stroke and Dementia Research (ISD), University Hospital, LMU Munich, Bavaria, Germany; ^4^ Department of Neurology, University Hospital, LMU Munich, Munich, Bavaria, Germany; ^5^ Max Planck School of Cognition, Leipzig, Sachsen, Germany; ^6^ German Center for Neurodegenerative Diseases (DZNE), Munich, Germany; ^7^ University Hospital, Ludwig‐Maximilians‐Universität, Munich, Germany; ^8^ Department of Neurology, Klinikum der Ludwig‐Maximilians Universität München, Munich, Bavaria, Germany; ^9^ University Hospital, LMU Munich, Munich, Germany; ^10^ Munich Cluster for Systems Neurology (SyNergy), Munich, Germany; ^11^ Munich Cluster for Systems Neurology (SyNergy), Munich, Bavaria, Germany; ^12^ Department of Neurology, University Hospital, LMU, Munich, Germany; ^13^ University Hospital, LMU Munich, Munich, Bavaria, Germany; ^14^ German Center for Neurodegenerative Diseases (DZNE), Munich, Bavaria, Germany; ^15^ Institute for Stroke and Dementia Research, LMU, Munich, Munich, Germany; ^16^ Department of Nuclear Medicine, University Hospital, LMU Munich, Munich, Germany, Munich, Germany; ^17^ Neurology, Klinikum der Ludwig‐Maximilians Universität München, Munich, Germany; ^18^ Department of Neurology, LMU University Hospital, LMU Munich, Munich, Munich, Germany; ^19^ Munich Cluster for Systems Neurology (SyNergy), Munich, Munich, Germany; ^20^ Life Molecular Imaging GmbH, Berlin, Germany; ^21^ Department of Laboratory Medicine and Pathobiology, University of Toronto, Toronto, ON, Canada; ^22^ Tanz Centre for Research in Neurodegenerative Diseases, University of Toronto, Toronto, ON, Canada; ^23^ Rossy PSP Program, University Health Network and the University of Toronto, Toronto, ON, Canada; ^24^ Department of Neurology, Klinikum der Ludwig‐Maximilians Universität München, Munich, Germany; ^25^ University of Gothenburg, The Sahlgrenska Academy, Institute of Neuroscience and Physiology, Psychiatry and Neurochemistry, Gothenburg, Sweden; ^26^ Institute for Stroke and Dementia Research (ISD), LMU University Hospital, LMU, Munich, Bavaria, Germany

## Abstract

**Background:**

Neurodegenerative 4‐repeat (4R) tauopathies commonly manifest as progressive supranuclear palsy (PSP). PSP patients show elevated PI‐2620‐PET in subcortical 4R tau predilection sites (e.g., globus pallidus), suggesting PI‐2620‐PET as a promising 4R tau neuroimaging candidate. However, optimal quantification of PI‐2620‐PET in 4R tauopathies remains challenging, as conventional cerebellar tau‐PET reference regions also accumulate 4R tau. We aimed to use unbiased image‐derived input function (IDIF) PET data to determine an optimized PET reference region for in vivo quantification of 4R tau.

**Methods:**

We obtained 60‐minute dynamic PI‐2620‐PET in 54 PSP Richardson Syndrome (PSP‐RS) patients and 19 healthy controls (HC), applying IDIF‐modeling using carotid timeseries to assess unbiased PI‐2620‐PET binding and determine total distribution volume (VT). Through an iterative approach, we intensity‐normalized VT‐images against white‐matter regions in the Hammers brain atlas, identifying regions where intensity‐normalized pallidum PET values showed the largest PSP‐RS vs. HC differences. White‐matter regions with strongest PSP‐RS vs. HC differences surviving multiple‐comparison correction were summarized into a single reference region spanning bilateral temporo‐orbital white‐matter. This ROI was then used to determine SUVRs using conventional 20‐40 minute PI‐2620‐PET data in PSP‐RS, a PSP‐non‐RS validation sample (*n* = 63), as well as non‐tau disease controls (i.e., alpha‐synucleinopathies, *n* = 20; Alzheimer's disease, *n* = 23).

**Results:**

Using PI‐2620 SUVRs obtained with the temporo‐orbital white‐matter reference, we detected strong PSP‐RS vs. HC group differences in basal ganglia SUVRs using voxel‐wise comparisons (*p* <0.001, FWE‐cluster corrected). Similar basal ganglia differences were detected for PSP‐non‐RS vs. HC, but not for alpha‐syn (no group differences) or AD vs. HC (cortical AD‐like group differences). In contrast, minimal group differences were found using a conventional inferior cerebellar grey matter reference region.

**Conclusions:**

Our findings strongly suggest temporo‐orbital white‐matter is superior to inferior cerebellum as a reference region for PI‐2620‐PET imaging in 4R tauopathies, due to increased sensitivity and purported specificity for 4R tau.